# Combined analysis of trabectome and phaco-trabectome outcomes by glaucoma severity

**DOI:** 10.12688/f1000research.8448.2

**Published:** 2016-06-27

**Authors:** Yalong Dang, Pritha Roy, Igor I. Bussel, Ralitsa T. Loewen, Hardik Parikh, Nils A. Loewen

**Affiliations:** 1 Department of Ophthalmology, School of Medicine, University of Pittsburgh, Pittsburgh, USA

**Keywords:** glaucoma, outflow, surgery, trabectome, ab interno trabeculectomy, disease index

## Abstract

Prior glaucoma severity staging systems were mostly concerned with visual field function and retinal nerve fiber layer, but did not include intraocular pressure or medications to capture resistance to treatment. We recently introduced a simple index that combines pressure, medications, and visual field damage and applied it to stratify outcomes of trabectome surgery. In the analysis presented here, we combined data of trabectome alone and trabectome with same session cataract surgery to increase testing power and chances of effect discovery. This microincisional glaucoma surgery removes the primary resistance to outflow in glaucoma, the trabecular meshwork, and has been mostly used in mild glaucoma. Traditional glaucoma surgeries have a relatively high complication rate and have been reserved for more advanced disease stages. In the analysis presented here we include our data of trabectome combined with cataract surgery. This is a common practice pattern as both occur in the same age group with increasing frequency. For patients in higher glaucoma index (GI) groups, the intraocular pressure (IOP) reduction was 2.34+/-0.19 mmHg more than those in a GI group one level lower while holding everything else constant. Those who had undergone trabectome combined with phacoemulsification had an IOP reduction that was 1.29+/-0.39 mmHg less compared to those with trabectome alone. No statistically significant difference was found between genders and age groups while holding everything else constant. Hispanics had a 3.81+/-1.08 mmHg greater IOP reduction. Pseudoexfoliation and steroid glaucoma patients had an IOP reduction that was greater by 2.91+/-0.56 and 3.86+/-0.81 mmHg, respectively, than those with primary open angle glaucoma. These results suggest a role for trabectome-mediated ab interno trabeculectomy beyond mild forms of glaucoma. Additionally, the multifactorial glaucoma index demonstrates a role in staging patients when comparing glaucoma surgical modalities.

## Introduction

Due to an increasing human lifespan, chronic diseases that manifest later in life, such as glaucoma and cataracts, have an increasing incidence and often occur in the same individuals ^1^. Medications ^2^ and surgeries ^3^ for glaucoma can induce or worsen cataracts. As a results, combining both cataract and glaucoma surgery is increasingly common, especially since the introduction of safer, microincisional glaucoma surgeries ^4, 5^. We recently examined the amount of intraocular pressure (IOP) reduction due to phacoemulsification at the time of trabectome surgery and found this to be clinically relatively insignificant ^6^. We also noted that trabectome surgery performed after failed trabeculectomy caused patients with more advanced visual field damage to have on average a greater pressure and medication reduction despite similar medications ^7^. In the current study we have consequently combined data of trabectome and phaco-trabectome surgery patients and stratified them by a glaucoma severity index. The idea behind developing a glaucoma index was to twofold: 1) to control for different indications for surgery and 2) to stratify outcomes by glaucoma severity.

Because indications for surgery may be a reduction of IOP, elimination of medications, or vision improvement either alone or, more commonly, a combination of those, we recently created a simple glaucoma index ^8^ that combines IOP, number of medications and visual field status to gauge relative clinical glaucoma severity but also describes resistance to treatment. Combining IOP and number of medications controls for treatment patterns. For instance, because glaucoma eye drops reduce IOP, a patient with an IOP of 16 mmHg on one medication might have a glaucoma that is clinically similarly challenging as one with an IOP of 24 mmHg off treatment. 

Plasma-mediated ab interno trabeculectomy with the trabectome, the surgery examined here, removes the nasal trabecular meshwork (TM) that is thought to be the primary outflow resistance in glaucoma ^9^. However, more recent insight has demonstrated a significant contribution by unidentified elements downstream of the trabecular meshwork ^6, 7, 10^ as half of the outflow resistance might remain unaccounted for ^11, 12^. Since removal of TM is achieved in all patients regardless of glaucoma severity, outcome differences have to reflect differences in the outflow tract distal to the TM. We included the visual field status to investigate whether IOP reduction from trabectome surgery might be correlated to glaucoma severity. By combining both trabectome and phaco-trabectome surgery data, a more complete picture can be obtained to guide surgeons on whether ab interno trabeculectomy may be an appropriate primary intervention.

## Methods

This retrospective analysis was approved (PRO14100026) by the Institutional Review Board of the University of Pittsburgh in accordance with the Declaration of Helsinki and the Health Insurance Portability and Accountability Act. Because of the retrospective nature, no consent was required. Glaucoma patients who received trabectome with or without phacoemulsification were enrolled, except in the following circumstances: history of glaucoma surgery, any subsequent cataract or glaucoma surgery in the follow-up period, and short term followup (less than 12 months). A history of laser trabeculoplasty did not lead to exclusion. Patients were divided into four groups (from mild to severe) according to a glaucoma index (GI), an indicator of glaucoma severity based on visual field, number of glaucoma medications, and preoperative IOP ^8^. Baseline IOP was divided into 4 groups, <20 mmHg, 20–29 mmHg, 30–39 mmHg, and above 40 and assigned with 1 to 4 points. Glaucoma medications (meds) were divided into 4 groups: ≤1, 2, 3, or ≥4, and assigned values of 1 to 4. Visual field (VF) was separated into 4 groups with points from 1 to 4: mild, moderate, advanced and end stage. GI was then defined as VF*meds*IOP and separated into GI group 1 = mild, GI group 2 = moderate, GI group 3 = advanced, and GI4 = severe defined based on glaucoma index scores of “≤4”, “4<;GI≤8”, “8<GI≤16”, and “>16,” respectively. The main outcome measure was the reduction of IOP. Secondary outcome measures included reduction of medication and a Kaplan-Meier survival analysis. Baseline characteristics were analyzed by the Kruskal-Wallis and chisquare tests for continuous and categorical variables between GI groups, respectively. Univariate linear regression was performed first and those demographics found to be statistically significant were included into the multivariate regression analysis. Kaplan-Meier was used for survival-curve analyses. Surgical success was defined as IOP≤21 mmHg or at least 20% IOP reduction from baseline in any two consecutive visits after three months and no secondary glaucoma surgery. Log-rank test was used to compare survival distributions of GI groups.

## Results

**Figure 1.**
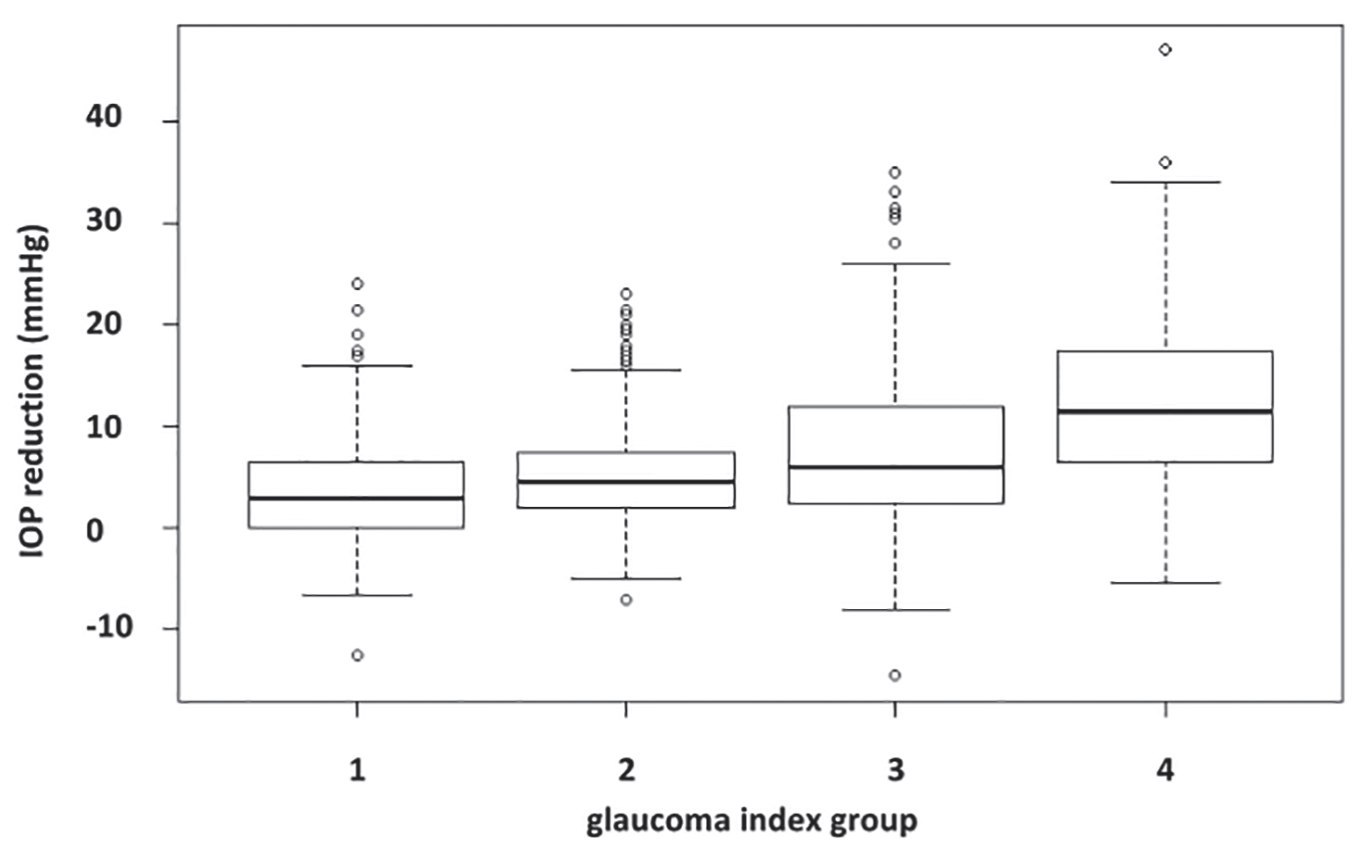
Reduction of intraocular pressure at 1 year. More severe glaucoma was associated with a larger pressure reduction.

**Figure 2.**
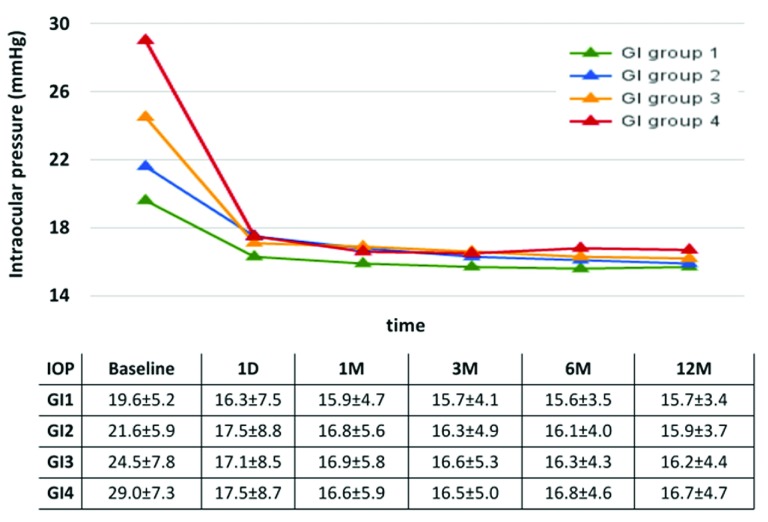
Intraocular pressure over time by glaucoma index group. Patients with a higher group had the largest decrease (average and standard deviation).

**Figure 3.**
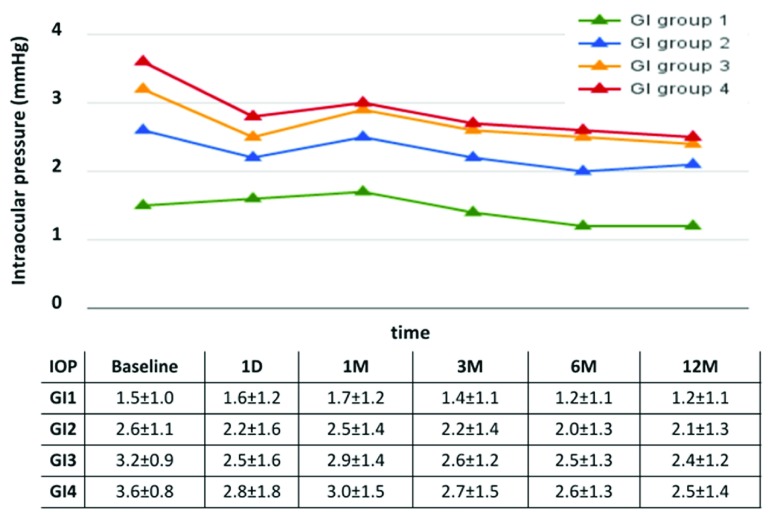
Medications by glaucoma index group. Patients in the severe and advanced groups had the largest medication reduction (average and standard deviation).

A total of 1340 cases of glaucoma patients were enrolled in the study and most of them were primary open angle glaucoma (POAG). The distribution across glaucoma severity groups was relatively even in number and average ages ( Table 1). There was a slight preponderance of female patients in the mild and moderate groups. The ethnicity of most patients was Caucasian followed by Asian. POAG and pseudoexfoliation glaucoma were the most common diagnoses. The cup disc ratio increased by glaucoma index group and more patients were phakic than pseudophakic. More patients in the higher GI groups had a trabectome surgery that was combined with cataract surgery. Patients with a higher GI group had a more profound IOP reduction ( Figure 1). At one year, the mean IOP reduction was 3.57±5.01, 5.34±5.40, 7.75±7.40, 12.09±8.08 mmHg for GI group 1 to 4, respectively. This pressure decrease occurred already on day 1 and remained relatively stable ( Figure 2). Similarly, patients with more severe glaucoma experienced a larger reduction in medications which were tapered more gradually ( Figure 3). When we stratified the overall IOP reduction by glaucoma severity, patients with worse glaucoma had the largest decrease.

**Table 1.** Demographics. Glaucoma index (GI); GI1 Mild: GI≤4; GI2 Moderate: 4<GI≤8; GI3 Advanced: 8<GI≤16; GI4 Severe: GI>16.

	GI Group 1 n=368	GI Group 2 n=322	GI Group 3 n=370	GI Group 4 n=280	p-value
**Age**					0.02
Mean±SD	71±11	68±13	69±15	66±18	
Range	18 – 92	18 – 90	18 – 96	18 – 96	
**Gender**					<0.01
Female	225 (61%)	181 (56%)	183 (49%)	135 (48%)	
Male	139 (38%)	139 (43%)	184 (50%)	136 (49%)	
NR	4 (1%)	2 (1%)	3 (1%)	9 (3%)	
**Race**					<0.01
African American	29 (8%)	16 (5%)	17 (5%)	14 (5%)	
Asian	98 (27%)	86 (27%)	115 (31%)	107 (38%)	
Caucasian	203 (55%)	177 (55%)	202 (55%)	124 (44%)	
Hispanic	23 (6%)	16 (5%)	17 (5%)	21 (8%)	
Other	15 (4%)	27 (8%)	19 (5%)	14 (5%)	
**Glaucoma Diagnosis**					<0.01
Primary open angle	288 (78%)	241 (75%)	257 (69%)	179 (64%)	
Pseudoexfoliation	28 (8%)	37 (11%)	59 (16%)	41 (15%)	
Pigment Dispersion	18 (5%)	10 (3%)	11 (3%)	6 (2%)	
Steroid	10 (3%)	18 (6%)	28 (8%)	39 (14%)	
Open angle type not specified	24 (7%)	16 (5%)	15 (4%)	15 (5%)	
**Visual Acuity (logMAR)**					<0.01
Mean±SD	0.31±0.34	0.28±0.35	0.37±0.52	0.47±0.61	
Range	-0.19 – 2.12	-0.19 – 2.00	-0.19 – 3.00	-0.19 – 3.00	
**Cup/Disc Ratio**					<0.01
Mean±SD	0.69±0.18	0.74±0.15	0.75±0.17	0.83±0.12	
Range3	0.1 – 1.9	0.3 – 1.00	0.1 – 1.00	0.25 – 1.00	
**Lens Status**					<0.01
Phakic	296 (80%)	231 (72%)	240 (65%)	159 (57%)	
Pseudophakic	56 (15%)	78 (24%)	116 (31%)	104 (37%)	
Aphakic	1 (0%)	1 (0%)	0 (0%)	3 (1%)	
Not recorded	15 (4%)	12 (4%)	14 (4%)	14 (5%)	
**Shaffer Grade**					0.47
I	5 (1%)	6 (2%)	4 (1%)	2 (1%)	
II	23 (6%)	25 (8%)	20 (5%)	27 (10%)	
III	107 (29%)	99 (31%)	121 (33%)	85 (30%)	
IV	192 (52%)	146 (45%)	172 (46%)	134 (48%)	
NR	41 (11%)	46 (14%)	53 (14%)	32 (11%)	
**Combined Surgeries**					<0.01
Trabectome+Phaco	164 (45%)	202 (63%)	260 (70%)	216 (77%)	
Trabectome Alone	204 (55%)	120 (37%)	110 (30%)	64 (23%)	

In the univariate regression analysis, age was slightly negatively correlated with the amount of IOP reduction ( Table 2) but this was not noted in the multivariate regression ( Table 3) while male gender had a positive correlation in the univariate but not anymore in the multivariate regression. For patients in the higher GI group, the IOP reduction was 2.34±0.19 mmHg more than those in one level lower GI group while holding everything else constant. Hispanics experienced a pressure drop larger by 3.81±1.08 mmHg than other ethnicities as did patients with a diagnosis of pseudoexfoliation and steroid induced glaucoma ( Table 3). IOP reduction was 2.91±0.56 and 3.86±0.81 mmHg more than in POAG patients. Interestingly, cataract surgery was associated with a slightly worse IOP reduction by 1.29+/-0.39 mmHg ( Table 3).

**Table 2.** Univariate regression.

	Coefficient	Standard Error	p-value
**Age**	-0.06	0.02	<0.01
**Male**	1.12	0.40	0.01
**Ethnicity**			
Asian	0.45	0.90	0.61
Caucasian	0.12	0.87	0.89
Hispanic	4.45	1.16	<0.01
Other	-0.57	1.26	0.66
**Diagnosis**			
Open angle type not specified	0.78	0.90	0.39
Pigmentary Dispersion	0.41	1.12	0.71
Pseudoexfoliation Glaucoma	3.44	0.59	<0.01
Steroid Glaucoma	5.38	0.88	<0.01
**Cup/Disc Ratio**	-2.30	1.34	0.09
**Shaffer Grade**	-0.38	0.31	0.21
**Lens**			
Aphakic	0.59	3.10	0.85
Pseudophakic	0.66	0.49	0.18
**Combined with phaco**	-3.20	0.41	<0.01

**Table 3.** Multivariate Regression.

	Coefficient	Standard Error	p-value
**Glaucoma index** **group**	2.34	0.19	<0.01
**Age**	-0.02	0.02	0.11
**Male**	0.60	0.38	0.12
**Ethnicity**			
Asian	-0.57	0.84	0.50
Caucasian	0.10	0.84	0.90
Hispanic	3.81	1.08	<0.01
Other	-0.30	1.05	0.78
**Diagnosis**			
Open angle type not specified	1.30	0.79	0.10
Pigmentary Dispersion	0.54	1.10	0.62
Pseudoexfoliative Glaucoma	2.91	0.56	<0.01
Steroid Glaucoma	3.86	0.81	<0.01
**Combined with** **phaco**	-1.29	0.39	<0.01

**Figure 4.**
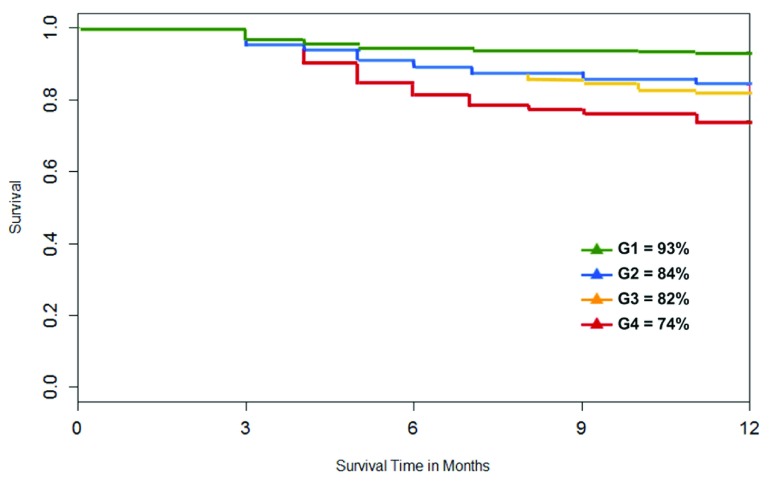
Survival plot by GI groups. The highest GI group with more advanced glaucoma (GI4, red) had the worst survival.

Survival rate at 12 months was 93%, 84%, 82% and 74% for GI group 1 to 4 ( Figure 4). Log-rank test indicated statistically significant differences between the GI groups and patients in the lower GI groups had a higher survival rate than those in higher GI groups.

## Discussion

The results of the current study are confirmatory of our prior study where we examined the impact of a glaucoma severity index on the results of trabectome surgery when done as a standalone procedure ^14^. The larger number of patients involved here allowed discovery of additional factors. We included here phaco-trabectome patients, who have a different, mixed indication that often includes visually significant cataract as the primary motivator while presenting with a relatively stable glaucoma. without the need for pressure reduction but a motivation to reduce medications. We did so after demonstrating by a rigorous statistical matching method, coarsened exact matching ^13^, that phacoemulsification does not contribute significantly to IOP reduction when done at the same time ^6^ or in a surgery prior to trabectome surgery ^14^. As in our prior studies ^14^, phaco-trabectome patients had a lower preoperative IOP. This study focused on stratification of outcomes by glaucoma index and did not apply advanced matching strategies that we applied elsewhere ^6, 14, 15^ to compare two groups. Hence, one cannot conclude from this study that cataract surgery is causatively linked to a diminished IOP reduction when compared to trabectome-only patients. On the contrary, an IOP reduction in many of our phaco-trabectome patients is simply not necessary to the same degree.

The results of this study match established risk factors and findings from other studies. Steroid glaucoma and pseudoexfoliation often produce very high IOPs and the primary pathology is located in the trabecular meshwork. As a result, ablating the meshwork reduces intraocular pressure very effectively ^17^.

In that study we found that a larger pressure reduction is achieved in more severe glaucoma consisting of a more advanced visual field damage, a higher pre-intervention IOP and more medications. We had previously found that cup disc ratio, Hispanic ethnicity and diagnosis of steroid-induced glaucoma are related to a larger IOP reduction ^14^. In addition to steroid induced glaucoma, pseudoexfoliation glaucoma confers a larger IOP reduction in the present study. Although phacoemulsification was negatively correlated with IOP reduction in this analysis, something that has been described for combined traditional trabeculectomy ^18, 19^, it is important to recall that this study was not designed to formally compare outcomes of combined versus trabectome-alone as we have done before ^9^. The number of patients analyzed here is significantly higher than in the two separate studies but we note similar results in the regression analysis allowing to discover additional factors.

The Goldmann equation describes that the limiting factor to IOP reduction after removal of the trabecular meshwork, the substrate of the main outflow resistance, is the episcleral venous pressure and uveoscleral outflow ^11^. The data presented here indicate that that this is mostly true also for more advanced glaucoma and consistent with two prior studies that demonstrated that significant conventional outflow can be recovered even after failed tube shunts ^20^ and after failed trabeculectomy ^10^. Small differences of the eventually achieved pressures could be explained by an episcleral venous pressures that is higher in glaucoma ^12^. This study has several limitations: to maximize patient number, we included all trabectome surgeries regardless of lens status or lens-cosurgery because our prior studies indicated that neither had a clinically relevant impact ^14, 22, 23^. This study was limited to only one year when follow up and data integrity was most amenable to automated retrieval and analysis. As a retrospective analysis, this study cannot discover causality and is only able to advise that patients with more severe glaucoma had a similar postoperative IOP and a comparable reduction in medications as those with mild glaucoma. Overall, the data presented here suggest that ab interno trabeculectomy might be an appropriate surgery to attempt to control more than mild glaucoma.

## Data availability

The data referenced by this article are under copyright with the following copyright statement: Copyright: © 2016 Dang Y et al.

The raw datasets could not be made available because the data could not be sufficiently anonymised to protect patient confidentiality. No individuals other than the investigators or research staff involved in the conduct of this research study and authorized representatives of the University Research Conduct and Compliance Office (RCCO) are permitted access to research data or documents (including medical record information) associated with the conduct of this research study. Institutional IRB rules are available on the following University of Pittsburgh OSIRIS website: http://www.osiris.pitt.edu/osiris. The approval permit number for this study is PRO14100026.
