# Supplementary material for: Combined analysis of trabectome and phaco-trabectome outcomes by glaucoma severity
Source: F1000Res. 2016 Jun 27;5:762. Originally published 2016 Apr 27. [Version 2] doi: 10.12688/f1000research.8448.2 (PMC4870993; doi:10.12688/f1000research.8448.2)
Supplement: Peer review history

# Peer review: Referee response for version 2

**DOI:** 10.5256/f1000research.9711.r14602

**Published:** 2016-06-27

**Authors:** William Myers

**Affiliations:** 1 Department of Ophthalmology, Feinberg School of Medicine, Northwestern University, Chicago, IL, USA

I have read this submission. I believe that I have an appropriate level of expertise to confirm that it is of an acceptable scientific standard.

# Peer review: Referee response for version 2

**DOI:** 10.5256/f1000research.9711.r14600

**Published:** 2016-06-27

**Authors:** Steven R. Sarkisian Jr.

**Affiliations:** 1 University of Oklahoma College of Medicine, Oklahoma City, OK, USA

I have read this submission. I believe that I have an appropriate level of expertise to confirm that it is of an acceptable scientific standard.

# Peer review: Referee response for version 1

**DOI:** 10.5256/f1000research.9096.r13594

**Published:** 2016-05-20

**Authors:** Steven R. Sarkisian Jr.

**Affiliations:** 1 University of Oklahoma College of Medicine, Oklahoma City, OK, USA

This is an interesting study demonstrating that ab interno trabeculectomy is an appropriate surgery to attempt to control more than mild glaucoma. Ab interno trabeculotomy is a growing trend in glaucoma surgery, not just with the trabectome device, but also with the dual blade that is recently on the market from New World Medical, and also the Sight Sciences device called TRAB 360. The trend toward ab interno, non-penetrating surgery in patients with not only mild, but even advanced glaucoma demonstrates our aversion for conventional filtration surgery and the significantly high complication rate seen with it. I am grateful to the authors for writing up their large case series of results that mirror a "real world" practice using this procedure. Glaucoma surgeons need to consider expanding the use of this type of technique, knowing that by doing so, they do not rule out the possibility of doing conventional surgery in the future, if necessary.

I have read this submission. I believe that I have an appropriate level of expertise to confirm that it is of an acceptable scientific standard.

# Peer review: Referee response for version 1

**DOI:** 10.5256/f1000research.9096.r13591

**Published:** 2016-05-13

**Authors:** William Myers

**Affiliations:** 1 Department of Ophthalmology, Feinberg School of Medicine, Northwestern University, Chicago, IL, USA

The concept ​of creating glaucoma index buckets ​has been validated by the authors​ ​in their ​

*PLoS O​ne *paper ^1^.  They showed that similar patients with and without cataract undergoing ab-interno trabeculectomy can be combined in one ​study. I ​found the​ conclusion that use of the Trabectome​ would be potentially useful in many cases of procedures combined with cataract surgery​,​ even ​when​ the glaucoma is fairly far advanced. As the site of greatest resistance to fluid exit, removing the trabecular membrane alone could potentially lower a high enough pressure gradient, at least temporarily. With the potential to avoid a full thickness filter and its attendant complications, the Trabectome could buy time to ride out a post operative cataract pressure spike.​

I would like to see more analysis of when an ab-interno trabeculectomy might be risky such as in severe glaucoma with ​split fixation where the alternative is ​an ab-externo​ filter or tube and ​then later cataract surgery. If this procedure is more effective than iStent and other bypass procedures, and involves no costly per case costs in comparison​,​ then Trabectome with phacoemulsification may be the most prudent choice​.

.  

​A​t the other end of the spectrum ​in ocular hypertension patients with no current measurable damage, ​is​ the risk profile ​of Trabectome combined with phacoemulsification reasonable​,​ compared to the risk and cost of drops or SLT as alternatives? We know that the effectiveness of only one iStent, the FDA approved norm in the US, is quite often not sufficient to reduce pressures adequately.​

There was another ​issue ​to be considered. The grading system us​es​ pressure as one of the components and also use​s the ​pressure drop as ​the​ metric of success. There be some circular reasoning involved​, a​s the initial pressure head itself is a determinant of ​the resulting ​pressure drop ​using​ drops, lasers ​or​ surgery. Unfortunately the study can not be replicated exactly due to patient confidentiality considerations.

​The validated glaucoma severity scale. from their previous paper in PLoS, uses simple-to-obtain inputs: IOP, number of medications and visual field loss; graded using MD and PSD from the visual field testing.​ The combination of these factors with definable numeric values can be utilized by cataract surgeons without glaucoma subspecialties.

I have read this submission. I believe that I have an appropriate level of expertise to confirm that it is of an acceptable scientific standard.

## References

[1] LoewenRTRoyPParikhHADangYSchumanJSLoewenNA: Impact of a Glaucoma Severity Index on Results of Trabectome Surgery: Larger Pressure Reduction in More Severe Glaucoma. *PLoS One*.2016;11(3) : 10.1371/journal.pone.0151926 e0151926 10.1371/journal.pone.0151926 27008637PMC4805208

# Response

**Published:** 2016-06-09

**Authors:** Nils Loewen

*I would like to see more analysis of when an ab-interno trabeculectomy might be risky such as in severe glaucoma with split fixation where the alternative is an ab-externo filter or tube and then later cataract surgery. If this procedure is more effective than iStent and other bypass procedures and involves no costly per case costs in comparison, then Trabectome with phacoemulsification may be the most prudent choice.*

**Authors: **This study is designed to analyze how patients with more severe glaucoma fared during the one-year follow-up, and the all-inclusive design maximizes the patient numbers. The results indicate that this microincisional surgery is a viable first option even in more severe glaucoma for many patients, but the resulting pressure is not low enough for many as the survival curve demonstrates. Postoperative complications are surprisingly common after traditional glaucoma surgery. Leakage, endophthalmitis, hypotony,  hardware erosion, damage to ocular tissues and other complications that are vision threatening occur with an additive probability of 77% in trabeculectomies and 58% in glaucoma drainage implants. ^9^ The surgeon has to discuss with each patient whether the avoidance of postoperative complications of traditional surgeries is worth the risk of an insufficient pressure reduction and the need to move on. In this context, it is important to recall that the postoperative IOP for tubes and trabeculectomies is only approximately 1 to 2 mmHg less that reported here. ^10^

 In forthcoming publications with fewer patients and full data disclosure, we report propensity score matched results of tube shunts and trabectome for a valid comparison of these surgical modalities. Because of the speed and safety of ab interno trabeculectomy, there is also a role for combining the two to achieve a lower IOP with fewer medications.

*At the other end of the spectrum in ocular hypertension patients with no current measurable damage, is the risk profile of Trabectome combined with phacoemulsification reasonable, compared to the risk and cost of drops or SLT as alternatives? We know that the effectiveness of only one iStent, the FDA approved norm in the US, is quite often not sufficient to reduce pressures adequately.*

**Authors: **It is hard to compare the effectiveness of different surgeries and in various practices without a randomized controlled design. The only way to obtain a high-quality comparison is to perform matching as we have done recently. ^3,5^ A cost analysis is similarly specific to the geographical area and practice. Prior cost analyses suggest that SLT is better or similar to eye drops in lowering IOP and more cost effective, even returning more than initially invested. ^11,12^ The same can be said about micro-incisional glaucoma surgeries ^13^ although this depends on the long-term viability of the chosen surgical modality and costs of anesthesia. SLT is our first line of treatment before use of drops and trabectome surgery presents a sharp step up. It is certainly convenient, fast and safe to combine at the time of cataract surgery. After trabectome surgery, only the distal outflow tract generates pressure causing most patients to drop into the mid-teens while SLT achieves more of a relative reduction that depends on the preoperative IOP. Our study presented here shows that the postoperative results are mostly independent of preoperative IOP (or that a larger IOP reduction is achieved in higher preoperative IOP).

*There was another issue to be considered. The grading system uses pressure as one of the components and also use s the pressure drop as the metric of success. There be some circular reasoning involved, as the initial pressure head itself is a determinant of the resulting pressure drop using drops, lasers or surgery.*

**Authors: **Different from other glaucoma staging systems graded by a defect of visual field or optic nerve injury ^14–16^, the glaucoma index here includes the relative resistance to treatment by individual patients. There is no circular logic, however, because we did not develop a prediction model that would have used a set of normal patients to train an algorithm and control patients within a study that were separated out from progressing patients as we have used elsewhere ^17^. Both IOP and medications are reduced (or one could say the glaucoma index is reduced) by this procedure.****

*Unfortunately the study can not be replicated exactly due to patient confidentiality considerations.*

**Authors:** HIPAA concerns are particularly relevant for this study describing many individuals. Fines can quickly climb to millions of dollars: https://www.truevault.com/blog/what-is-the-penalty-for-a-hipaa-violation.html We are very impressed with F1000Research and the compelling benefits of pre-printing and open peer review and will provide full open data with our upcoming publications. Although it is the standard not to release full datasets in clinical ophthalmology publications, we have come to realize that this is a disservice. For instance, one could apply matching strategies to other datasets to compare surgeries and we hope to make ours available for this purpose.

*The validated glaucoma severity scale from their previous paper in PLoS, uses simple-to-obtain inputs: IOP, the number of medications and visual field loss graded using MD and PSD from the visual field testing. The combination of these factors with definable numeric values can be utilized by cataract surgeons without glaucoma subspecialties.*

**Authors:** Yes, we agree. We were surprised that this has not been attempted before. Hopefully, better indices will become available. We have a glaucoma risk calculator in our medical record system, a customized version of Epic, and that has been a big step forward.

**References used in this Reply to Reviewers**

1. Loewen, R. T. et al. Impact of a Glaucoma Severity Index on Results of Trabectome Surgery: Larger Pressure Reduction in More Severe Glaucoma. PLoS One 11, e0151926 (2016).
2. Bussel, I. I., Kaplowitz, K., Schuman, J. S., Loewen, N. A. & Trabectome Study Group. Outcomes of ab interno trabeculectomy with the trabectome by degree of angle opening. Br. J. Ophthalmol. 99, 914–919 (2015).
3. Parikh, H. A., Bussel, I. I., Schuman, J. S., Brown, E. N. & Loewen, N. A. Coarsened Exact Matching of Phaco-Trabectome to Trabectome in Phakic Patients: Lack of Additional Pressure Reduction from Phacoemulsification. PLoS One 11, e0149384 (2016).
4. Wollstein, G. et al. Retinal nerve fibre layer and visual function loss in glaucoma: the tipping point. Br. J. Ophthalmol. 96, 47–52 (2012).
5. Neiweem, A. E., Bussel, I. I., Schuman, J. S., Brown, E. N. & Loewen, N. A. Glaucoma Surgery Calculator: Limited Additive Effect of Phacoemulsification on Intraocular Pressure in Ab Interno Trabeculectomy. PLoS One 11, e0153585 (2016).
6. Iacus, S. M., King, G. & Porro, G. Causal Inference without Balance Checking: Coarsened Exact Matching. Polit. Anal. 20, 1–24 (2012).
7. Parikh, H. A., Bussel, I. I., Schuman, J. S., Brown, E. N. & Loewen, N. A. Coarsened Exact Matching of Phaco-Trabectome to Trabectome in Phakic Patients: Lack of Additional Pressure Reduction from Phacoemulsification. PLoS One 11, e0149384 (2016).
8. Akil, H. et al. Clinical Results of Ab Interno Trabeculotomy Using the Trabectome in Patients with Pigmentary Glaucoma compared to Primary Open Angle Glaucoma. Clin. Experiment. Ophthalmol. (2016). doi: 10.1111/ceo.12737
9. Gedde, S. J. et al. in Am J Ophthalmol 153, 804–814.e1 (2012 Elsevier Inc, 2012).
10. Gedde, S. J. et al. Treatment Outcomes in the Tube Versus Trabeculectomy (TVT) Study After Five Years of Follow-up. Am. J. Ophthalmol. 153, 789–803.e2 (2012).
11. Taylor, H. R. Glaucoma: where to now? Ophthalmology 116, 821–822 (2009).
12. Rein, D. B. et al. The cost-effectiveness of routine office-based identification and subsequent medical treatment of primary open-angle glaucoma in the United States. Ophthalmology 116, 823–832 (2009).
13. Iordanous, Y., Kent, J. S., Hutnik, C. M. & Malvankar-Mehta, M. S. Projected Cost Comparison of Trabectome, iStent, and Endoscopic Cyclophotocoagulation Versus Glaucoma Medication in the Ontario Health Insurance Plan. J. Glaucoma (2013). doi: 10.1097/IJG.0b013e31829d9bc7
14. Brusini, P. & Filacorda, S. Enhanced Glaucoma Staging System (GSS 2) for classifying functional damage in glaucoma. J. Glaucoma 15, 40–46 (2006).
15. Mills, R. P. et al. Categorizing the stage of glaucoma from pre-diagnosis to end-stage disease. Am. J. Ophthalmol. 141, 24–30 (2006).
16. Advanced Glaucoma Intervention Study. 2. Visual field test scoring and reliability. Ophthalmology 101, 1445–1455 (1994).
17. Zhang, X. et al. Predicting Development of Glaucomatous Visual Field Conversion Using Baseline Fourier-Domain Optical Coherence Tomography. Am. J. Ophthalmol. 163, 29–37 (2016).

# Peer review: Referee response for version 1

**DOI:** 10.5256/f1000research.9096.r13590

**Published:** 2016-05-10

**Authors:** Kristy G. Mascarenhas

**Affiliations:** 1 Hartford Hospital/Middlesex Hospital, Middletown, CT, USA

This was an interesting analysis of the use of Trabectome surgery in patients with more severe glaucomas. Comments and questions are as follows, most of which would clarify the methods and data analysis for the reader:

- The abstract and introduction could be clearer about the purpose of the study.  You have to wait a long time to find out that this was a retrospective chart review of patients who had received Trabectome and phaco-Trabectome surgeries.  Were the authors looking for statistically significant differences in IOP reduction between the glaucoma severity groups, or equivalent effect in mild vs. severe glaucoma?  If so, was a power calculation done?
- The abstract makes it sound as though only phaco-Trabectome cases were included, but the introduction states that both Trabectome and combined surgeries were included. Please clarify the abstract?
- The methods section briefly introduces the glaucoma index system that was used, but especially for someone who has not read the previous article evaluating it, a bit more detail about the point system used would be helpful.
- One of the exclusion criteria was prior glaucoma surgery. Is data available about the number of patients who had previously had SLT/ALT?
- Table 1 shows the number of Trabectomes vs. phaco/Trabectomes. Did all phakic patients have cataract surgery, or did any receive Trabectome alone?
- Over what time period did the reviewed patients receive Trabectome? Relatively recently, or closer to the beginning of Trabectome use? How many different surgeons' patients were reviewed? (Is it possible that surgical techniques evolved over time, or that different surgeons' techniques varied significantly?)  A sentence like "We reviewed the charts of XXX patients from XXX surgeons who underwent CE/Trabectome or Trabectome alone between 2012-2016" would be very helpful.
- The authors state that patients in the severe GI group and patients with more severe glaucomas like pseudoexfoliation and steroid-response had a greater IOP reduction than other groups. Since IOP is one component of the glaucoma index and Trabectome does lower IOP more significantly with higher pre-op IOP, is it possible to say that Trabectome had a greater effect in these groups independently of pre-op IOP? (To confirm, was this analysis done?)
- Can the authors include a short discussion of limitations of the current study and possibly a bit of speculation on the smaller IOP reduction with phaco/Trabectome than with Trabectome alone, as well as on why the more severe GI groups saw a shorter-lived IOP reduction?

I have read this submission. I believe that I have an appropriate level of expertise to confirm that it is of an acceptable scientific standard.

# Response

**Published:** 2016-06-09

**Authors:** Nils Loewen

*This was an interesting analysis of the use of Trabectome surgery in patients with more severe glaucomas. Comments and questions are as follows, most of which would clarify the methods and data analysis for the reader: The abstract and introduction could be clearer about the purpose of the study.  You have to wait a long time to find out that this was a retrospective chart review of patients who had received Trabectome and phaco-Trabectome surgeries.*

1. *Were the authors looking for statistically significant differences in IOP reduction between the glaucoma severity groups, or equivalent effect in mild vs. severe glaucoma?  *
2. *If so, was a power calculation done?*

**Authors:** Thank you for the opportunity to revise our abstract and introduction.

 We have added to the abstract: “In the analysis presented here, we combined data of trabectome alone and trabectome with same session cataract surgery to increase testing power and chances of effect discovery.”

 We have also completely rewritten the introduction to explain  the purpose of combining trabectome and phaco-trabectome data better and the idea behind the glaucoma index. Briefly, we recently created a simple glaucoma index that combines IOP, the number of medications and visual field status to gauge relative clinical glaucoma severity but also describes resistance to treatment. This may control for medication use when trying to gauge IOP results and allow to focus on glaucoma severity related outcomes. We combined trabectome and phaco-trabectome data sets because phacoemulsification is not a significant contributor to IOP reduction in studies with advanced matching strategies.

 A power calculation was done and our regression analysis has a power of 0.95.  We would require 82 patients to detect a GI IOP difference for a medium sized effect of 0.3 and a power of 80%. As our statistician advises, a power analysis is only needed when no statistical significance is found and that is why we had not reported it in our original submission. If a statistically significant difference is found as here, then that suggests that the statistical test is powerful enough.

*The abstract makes it sound as though only phaco-Trabectome cases were included, but the introduction states that both Trabectome and combined surgeries were included. Please clarify the abstract?*

**Authors:** Thank you for noticing this. Both were included and we have added the following to the abstract: “In the analysis presented here, we combined data of trabectome alone and trabectome with same session cataract surgery to increase testing power and chances of effect discovery.”

*The methods section briefly introduces the glaucoma index system that was used, but especially for someone who has not read the previous article evaluating it, a bit more detail about the point system used would be helpful.*

**Authors:** We have updated our methods section to explain the individual factors in more detail: “Patients were divided into four groups (from mild to severe) according to a glaucoma index (GI), an indicator of glaucoma severity based on visual field, the number of glaucoma medications, and preoperative IOP ^1^. Baseline IOP was divided into 4 groups, <20 mmHg, 20-29 mmHg, 30-39 mmHg, and above 40 and assigned with 1 to 4 points. Glaucoma medications (meds) were divided into 4 groups: ≤1, 2, 3, or ≥4, and assigned values of 1 to 4. Visual field (VF) was separated into 4 groups with points from 1 to 4: mild, moderate, advanced and end-stage. GI was then defined as VF*meds*IOP and separated into GI group 1 = mild, GI group 2 = moderate, GI group 3 = advanced, and GI4 = severe defined based on glaucoma index scores of “≤4”, “4<GI≤8”, “8<GI≤16”, and “>16,” respectively.”

*One of the exclusion criteria was prior glaucoma surgery. Is data available about the number of patients who had previously had SLT/ALT?*

 Authors: Laser trabeculoplasty is the first line of treatment in our practice pattern, and this was not an exclusion criterion. Nearly all patients will have had this. Trabeculoplasty was not included as a factor in the analysis to reduce complexity. We have added to Methods: “A history of laser trabeculoplasty did not lead to exclusion.”

*Table 1 shows the number of Trabectomes vs. phaco/Trabectomes. Did all phakic patients have cataract surgery, or did any receive Trabectome alone?*

**Authors: **We have analyzed the effect of trabectome in phakic patients before in two different studies ^2,3^ and found that even in narrow angles there does not seem to be a significant difference. Consequently, we included here all patients, including trabectome patients who remained phakic, to focus on the glaucoma index without further break down into subgroups.

 It is important to recall that differences in phaco versus no phaco are merely based on averages. Because of a mixed indication for these patients (many cataract surgery patients do not need a pressure reduction and merely want to reduce medications) and because no formal matching was done, one cannot conclude from this study that cataract surgery is causatively linked to a diminished IOP reduction when compared to trabectome-only patients. On the contrary, an IOP reduction in many of our phaco-trabectome patients is simply not necessary to the same degree.

*Over what time period did the reviewed patients receive Trabectome? Relatively recently, or closer to the beginning of Trabectome use? How many different surgeons' patients were reviewed? (Is it possible that surgical techniques evolved over time, or that different surgeons' techniques varied significantly?)  A sentence like "We reviewed the charts of XXX patients from XXX surgeons who underwent CE/Trabectome or Trabectome alone between 2012-2016" would be very helpful.*

**Authors: **The study period included data from 2011 up until the end of 2015. We did not analyze outcomes by individual surgeons. We concede that a majority of surgeries was performed by the senior author of this study and after 2012. Unfortunately, is not easily possible to provide the exact breakdown due to how data is extracted from the EMR data cloud. Manual curation of thousands of patient operative reports would be necessary.

*the authors state that patients in the severe GI group and patients with more severe glaucomas like pseudoexfoliation and steroid-response had a greater IOP reduction than other groups. Since IOP is one component of the glaucoma index and Trabectome does lower IOP more significantly with higher pre-op IOP, is it possible to say that Trabectome had a greater effect in these groups independently of pre-op IOP? (To confirm, was this analysis done?)*

**Authors:** ****Thank you very much for this inquiry. Without conducting a prospective, randomized controlled study or applying an advanced matching strategy, it is not possible to detect a strong, likely causative, correlation between glaucoma type and the amount of resulting IOP drop. The multivariate regression analysis we have done here does show that pseudoexfoliation and steroid glaucoma are significantly associated with a larger IOP reduction. This would be expected since both are obvious trabecular meshwork diseases. It is not clear what the substrate is that prevents IOP from  reaching the theoretical limit of episcleral venous pressure after trabecular ablation or bypass.

 The glaucoma index already captures resistance to medical treatment and these eyes require an IOP that is considerably lower than what can be achieved by trabecular ablation. This may reflect a tipping point. ^4^

*Can the authors include a short discussion of limitations of the current study and possibly a bit of speculation on the smaller IOP reduction with phaco/Trabectome than with Trabectome alone, as well as on why the more severe GI groups saw a shorter-lived IOP reduction?*

**Authors:** ****Thank you. We would like to add to the discussion the following: “This study has several limitations: to maximize patient number, we included all trabectome surgeries regardless of lens status or lens-cosurgery because our prior studies indicated that neither had a clinically relevant impact. ^2,3,5^ This study was limited to only one year when follow-up and data integrity was most amenable to automated retrieval and analysis. As a retrospective analysis, this study cannot discover causality and is only able to advise that patients with more severe glaucoma had a similar postoperative IOP and a comparable reduction in medications as those with mild glaucoma.”

 We have also added a better explanation why phaco-trabectome patients have a lesser IOP reduction: “We included here phaco-trabectome patients, who have a different, mixed indication that often includes a visually significant cataract as the primary motivator while presenting with a relatively stable glaucoma without the need for pressure reduction but a motivation to reduce medications. We did so after demonstrating by a rigorous statistical matching method, coarsened exact matching ^6^, that phacoemulsification does not contribute significantly to IOP reduction when done at the same time ^7^ or in a surgery prior to trabectome surgery ^5^. As in our prior studies ^5^, phaco-trabectome patients had a lower preoperative IOP. This study focused on stratification of outcomes by glaucoma index and did not apply advanced matching strategies that we applied elsewhere ^5,7,8^ to compare two groups. Hence, one cannot conclude from this study that cataract surgery is causatively linked to a diminished IOP reduction when compared to trabectome-only patients. On the contrary, an IOP reduction in many of our phaco-trabectome patients is simply not necessary to the same degree.”

**References used in this Reply to Reviewers**

1. Loewen, R. T. et al. Impact of a Glaucoma Severity Index on Results of Trabectome Surgery: Larger Pressure Reduction in More Severe Glaucoma. PLoS One 11, e0151926 (2016).
2. Bussel, I. I., Kaplowitz, K., Schuman, J. S., Loewen, N. A. & Trabectome Study Group. Outcomes of ab interno trabeculectomy with the trabectome by degree of angle opening. Br. J. Ophthalmol. 99, 914–919 (2015).
3. Parikh, H. A., Bussel, I. I., Schuman, J. S., Brown, E. N. & Loewen, N. A. Coarsened Exact Matching of Phaco-Trabectome to Trabectome in Phakic Patients: Lack of Additional Pressure Reduction from Phacoemulsification. PLoS One 11, e0149384 (2016).
4. Wollstein, G. et al. Retinal nerve fibre layer and visual function loss in glaucoma: the tipping point. Br. J. Ophthalmol. 96, 47–52 (2012).
5. Neiweem, A. E., Bussel, I. I., Schuman, J. S., Brown, E. N. & Loewen, N. A. Glaucoma Surgery Calculator: Limited Additive Effect of Phacoemulsification on Intraocular Pressure in Ab Interno Trabeculectomy. PLoS One 11, e0153585 (2016).
6. Iacus, S. M., King, G. & Porro, G. Causal Inference without Balance Checking: Coarsened Exact Matching. Polit. Anal. 20, 1–24 (2012).
7. Parikh, H. A., Bussel, I. I., Schuman, J. S., Brown, E. N. & Loewen, N. A. Coarsened Exact Matching of Phaco-Trabectome to Trabectome in Phakic Patients: Lack of Additional Pressure Reduction from Phacoemulsification. PLoS One 11, e0149384 (2016).
8. Akil, H. et al. Clinical Results of Ab Interno Trabeculotomy Using the Trabectome in Patients with Pigmentary Glaucoma compared to Primary Open Angle Glaucoma. Clin. Experiment. Ophthalmol. (2016). doi: 10.1111/ceo.12737
9. Gedde, S. J. et al. in Am J Ophthalmol 153, 804–814.e1 (2012 Elsevier Inc, 2012).
10. Gedde, S. J. et al. Treatment Outcomes in the Tube Versus Trabeculectomy (TVT) Study After Five Years of Follow-up. Am. J. Ophthalmol. 153, 789–803.e2 (2012).
11. Taylor, H. R. Glaucoma: where to now? Ophthalmology 116, 821–822 (2009).
12. Rein, D. B. et al. The cost-effectiveness of routine office-based identification and subsequent medical treatment of primary open-angle glaucoma in the United States. Ophthalmology 116, 823–832 (2009).
13. Iordanous, Y., Kent, J. S., Hutnik, C. M. & Malvankar-Mehta, M. S. Projected Cost Comparison of Trabectome, iStent, and Endoscopic Cyclophotocoagulation Versus Glaucoma Medication in the Ontario Health Insurance Plan. J. Glaucoma (2013). doi: 10.1097/IJG.0b013e31829d9bc7
14. Brusini, P. & Filacorda, S. Enhanced Glaucoma Staging System (GSS 2) for classifying functional damage in glaucoma. J. Glaucoma 15, 40–46 (2006).
15. Mills, R. P. et al. Categorizing the stage of glaucoma from pre-diagnosis to end-stage disease. Am. J. Ophthalmol. 141, 24–30 (2006).
16. Advanced Glaucoma Intervention Study. 2. Visual field test scoring and reliability. Ophthalmology 101, 1445–1455 (1994).
17. Zhang, X. et al. Predicting Development of Glaucomatous Visual Field Conversion Using Baseline Fourier-Domain Optical Coherence Tomography. Am. J. Ophthalmol. 163, 29–37 (2016).
